# *Eimeria* species occurrence varies between geographic regions and poultry production systems and may influence parasite genetic diversity

**DOI:** 10.1016/j.vetpar.2016.12.003

**Published:** 2017-01-15

**Authors:** B. Chengat Prakashbabu, V. Thenmozhi, G. Limon, K. Kundu, S. Kumar, R. Garg, E.L. Clark, A.S.R. Srinivasa Rao, D.G. Raj, M. Raman, P.S. Banerjee, F.M. Tomley, J. Guitian, D.P. Blake

**Affiliations:** a Department of Production and Population Health, Royal Veterinary College, North Mymms, Hertfordshire, UK; b Department of Veterinary Parasitology, Madras Veterinary College, Tamil Nadu Veterinary and Animal Sciences University, Chennai, India; c Division of Parasitology, Indian Veterinary Research Institute, Izatnagar, Uttar Pradesh, India; d Department of Pathology and Pathogen Biology, Royal Veterinary College, North Mymms, Hertfordshire, UK; e Augusta University, Augusta, GA, USA; f Department of Animal Biotechnology, Madras Veterinary College, Tamil Nadu Veterinary and Animal Sciences University, Chennai, India

**Keywords:** *Eimeria*, Chickens, Epidemiology, Genetic diversity

## Abstract

Coccidiosis is one of the biggest challenges faced by the global poultry industry. Recent studies have highlighted the ubiquitous distribution of all *Eimeria* species which can cause this disease in chickens, but intriguingly revealed a regional divide in genetic diversity and population structure for at least one species, *Eimeria tenella*. The drivers associated with such distinct geographic variation are unclear, but may impact on the occurrence and extent of resistance to anticoccidial drugs and future subunit vaccines. India is one of the largest poultry producers in the world and includes a transition between *E. tenella* populations defined by high and low genetic diversity. The aim of this study was to identify risk factors associated with the prevalence of *Eimeria* species defined by high and low pathogenicity in northern and southern states of India, and seek to understand factors which vary between the regions as possible drivers for differential genetic variation. Faecal samples and data relating to farm characteristics and management were collected from 107 farms from northern India and 133 farms from southern India. Faecal samples were analysed using microscopy and PCR to identify *Eimeria* occurrence. Multiple correspondence analysis was applied to transform correlated putative risk factors into a smaller number of synthetic uncorrelated factors. Hierarchical cluster analysis was used to identify poultry farm typologies, revealing three distinct clusters in the studied regions. The association between clusters and presence of *Eimeria* species was assessed by logistic regression. The study found that large-scale broiler farms in the north were at greatest risk of harbouring any *Eimeria* species and a larger proportion of such farms were positive for *E. necatrix*, the most pathogenic species. Comparison revealed a more even distribution for *E. tenella* across production systems in south India, but with a lower overall occurrence. Such a polarised region- and system-specific distribution may contribute to the different levels of genetic diversity observed previously in India and may influence parasite population structure across much of Asia and Africa. The findings of the study can be used to prioritise target farms to launch and optimise appropriate anticoccidial strategies for long-term control.

## Highlights

• Multivariate analysis revealed comparable poultry clusters in north and south India.
• *Eimeria* species occurrence varied between system clusters.
• *E. tenella* occurrence across systems may underpin region-specific genetic diversity.
• *E. necatrix* was found to be more common in north than south India.

## 1 Introduction

Global poultry production has increased dramatically in the last 20 years with more than 90 million tonnes of chicken meat and 1.1 trillion eggs now produced every year (Blake and Tomley, 2014, FAOstat, 2015). Production is expected to continue to increase over the coming decades with South and East Asia, including India, major hotspots for expansion (Grace et al., 2012). As poultry production gains in significance, pathogens which can compromise efficient production and animal welfare become increasingly important. *Eimeria*, protozoan parasites within the phylum Apicomplexa, cause the disease coccidiosis in all livestock including chickens. Seven species, *Eimeria acervulina*, *E. brunetti*, *E. maxima*, *E. mitis*, *E. necatrix*, *E. praecox* and *E. tenella*, are known to infect the domestic chicken (*Gallus gallus domesticus*). *Eimeria brunetti*, *E. necatrix* and *E. tenella* are associated with haemorrhagic coccidiosis and can be highly pathogenic, with high morbidity and mortality possible (Long et al., 1976). The remaining four species are usually less pathogenic, incurring malabsorptive pathologies, although morbidity and mortality can occur depending on dose ingested, parasite strain-specific variation in virulence and host factors such as age, breed and immune status (Long et al., 1976, Williams et al., 2009). Co-infection with multiple species is common and can complicate diagnosis (Williams et al., 1996). *Eimeria necatrix* has been recognised as the most pathogenic *Eimeria* species which infects chickens, but *E. tenella* is more common and exerts a greater impact on poultry production (Long et al., 1976, Blake et al., 2015).

In recent studies of *E. tenella* occurrence around the world, the parasite was found to be common in all regions that were sampled (Clark et al., 2016). However, detailed investigation of *E. tenella* recovered from Egypt, India, Libya and Nigeria, demonstrated dramatically different levels of genetic diversity and population structures between regions (Blake et al., 2015). Comparison of parasites from North Africa and northern India with those from Nigeria and southern India revealed a geographic split between high and low diversity populations. The variation detected within India identified the country as potentially informative, possibly illustrating parameters with relevance to diversity in other Asian and African countries. *Eimeria tenella* collected from Haryana, Punjab, Uttarakhand and Uttar Pradesh in northern India segregated into a limited number of haplotypes, each of which were common. In direct contrast, *E. tenella* collected from Andhra Pradesh, Karnataka, Kerala, and Tamil Nadu in southern India were far more polymorphic with a large number of unique haplotypes detected, indicative of greater genetic mixing and cross-fertilisation between parasites (Blake et al., 2015). No parasite haplotypes detected in northern India were shared with the chicken population sampled in the south. Linkage disequilibrium was detected in northern, but not southern India supporting a conclusion that there are distinct drivers associated with this strong regional variation. Identification of these drivers can play a fundamental role in understanding, and possibly even controlling, the appearance of genetic diversity for *Eimeria* with relevance to the application and longevity of chemical and vaccinal anticoccidial control.

India is now one of the largest producers of poultry and poultry products in the world, providing more than 2.6 million tonnes of meat and 69.7 billion eggs in 2013 with average increases of 7.3 and 5.5% each year since 1993 (FAOstat, 2015). Chicken breeds reared in India include global commercial broiler and layer types comparable to those reared in Europe and North America (reared for meat and egg production respectively), supplemented by a growing population of indigenous birds and hybrids derived from crossbreeding commercial and indigenous lines (Khan, 2008). Indian breeds such as Aseel and Kadaknath are becoming increasingly popular as pure and out-crossed lines following perceived benefits including production traits and resistance to disease (Ramasamy et al., 2010, Arora et al., 2011, Haunshi et al., 2011). Poultry production systems in India are highly varied, including large-scale commercial farms, traditional small-scale farms and backyard stock keepers. Chickens are also reared for different purposes including meat, eggs and use as currency. Each system varies in underlying management practices and may have a different risk of pathogen exposure.

Previous studies have identified some management practises that predispose towards *Eimeria* occurrence, and thus enhance likelihood of disease as well as co-infection/cross-fertilisation. These have highlighted the importance of farm hygiene, choice of disinfection procedure, use of overalls and previous farm history of coccidiosis outbreaks (Graat et al., 1998, Al-Natour et al., 2002, Gharekhani et al., 2014). Sub-optimal control by anticoccidial drugs, either through their ineffective application or parasite genetic resistance, increases *Eimeria* occurrence, burden and disease (Chapman et al., 2010). Management strategies which disrupt the eimerian faecal-oral lifecycle, such as housing poultry on raised wired floors, reduce *Eimeria* occurrence and outbreaks of disease (Lunden et al., 2000). Chicken breed and age at slaughter also influence *Eimeria* occurrence (Bumstead and Millard, 1992, Shirzad et al., 2011). The occurrence of coccidiosis can also vary due to climatic conditions, with evidence of elevated parasite levels and disease during wetter and warmer seasons (Awais et al., 2012, Jatau et al., 2012, Luu et al., 2013).

As the scale and importance of poultry production intensifies in tropical regions, providing food security to a growing human population, it is important to understand the factors that influence the presence or absence of *Eimeria*. Determining drivers for the occurrence of each *Eimeria* species is also important as they vary in pathogenicity, and hence can have varying impact on farm productivity. Identifying typologies of farms based on the risk posed by *Eimeria* is also essential for future control strategies. Building on earlier studies focused on specific towns or states (e.g. Jithendran, 2001, Sharma et al., 2009, Aarthi et al., 2010, Jadhav et al., 2011) we have sampled chicken farms from eight states across northern and southern India to identify and compare farm risk factors associated with the presence of *Eimeria* and which might impact on genetic diversity.

## 2 Materials and methods

### 2.1 Ethical review

The questionnaire and environmental sampling protocols applied here were approved by the Royal Veterinary College Ethical Review Committee and assigned the reference URN 2014 1280. Farmers were asked for their signed consent to take part in the study before or at the start of each farm visit.

### 2.2 Study settings

#### 2.2.1 Study design

The study was carried out in eight states, including four from each of northern and southern India. The northern region covered the Haryana, Punjab, Uttarakhand and Uttar Pradesh states. The southern region involved Andhra Pradesh, Karnataka, Kerala and Tamil Nadu (including Pondicherry). Production systems in south India were likely to be more industrialised with a greater emphasis on broiler production (Mehta and Nambiar, 2007). The states sampled from the north have humid sub-tropical (warm summer) and semi-arid climates. The states sampled in the south have tropical wet, tropical wet and dry, and semi-arid climates (National Building Code of India; https://law.resource.org/pub/in/bis/S03/is.sp.7.2005.pdf).

**Fig. 1:**
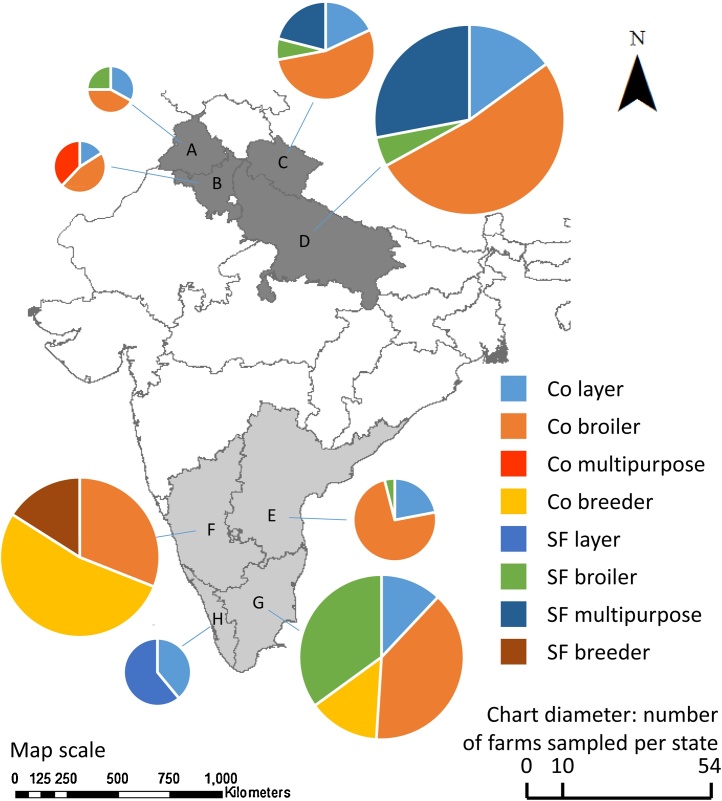
Map showing part of India indicating the number and type of poultry units sampled in northern and southern India. The map does not represent political boundaries. The diameter of each pie chart indicates the number of farms sampled from each state. A = Punjab, B = Haryana, C = Uttarakhand, D = Uttar Pradesh, E = Andhra Pradesh, F = Karnataka, G = Tamil Nadu (including Pondicherry), H = Kerala. Co = commercial, SF = small flock (including backyard and small scale indigenous production).

In each state a list of poultry farms was obtained from poultry disease diagnostic laboratories, veterinary outlets and commercial poultry producers. Based on these lists farms were selected for sampling within each state. The data were collected as part of a parasite genetic diversity assessment which was deliberately designed to sample from as wide a range of backgrounds as possible. However, for practical reasons related primarily to accessibility most commercial broiler farms sampled reared birds under contract with either Venketeshwara Hatcheries Private Ltd. or Suguna Poultry Farms Ltd. Combined, these two companies share more than 40% of the total poultry production in India and were the dominant producers in the regions sampled. Farms less than 100 m away from a previously sampled farm were excluded, as were farms with shared staff or equipment. A minimum target number of 100 farms in each region was established as it was considered to be logistically feasible and likely to adequately capture the variability of the production systems in the two areas. No formal sample calculation was conducted. The study sample consisted of 107 farms from north India and 133 farms from south India for which complete questionnaire and *Eimeria* test results were available. No farmers refused to participate. The distribution of farms sampled is presented in Fig. 1. All farms were visited between October 2010 and February 2014. Researcher teams from the northern and southern regions shared standardised protocols and undertook laboratory exchanges of personnel to ensure strict protocol adherence and minimise the risk of researcher bias.

#### 2.2.2 Faecal sample collection and analysis

Faecal droppings were collected in 50 ml polypropylene Falcon tubes prefilled with 5 ml 2% (w/v) potassium dichromate as described previously (Kumar et al., 2014). Briefly, between three and five tubes were filled from a single pen per farm by walking a predetermined “W” pathway and collecting one fresh dropping every two to five paces, providing a semi-randomised approach to sampling and minimising the risk of unconscious bias. In farms with multiple pens one pen was selected without pre-defined formal selection criteria, but trying to provide a sample representative of the farm. Thus, each sample represented multiple birds within a single cohort on a single farm. A farm was considered positive for an *Eimeria* species if at least one tube was found to be positive.

All faecal samples were tested for the presence of *Eimeria* oocysts microscopically as described previously, with no attempt to determine species identity by oocyst morphology (Kumar et al., 2014). Instead, PCR was used to determine *Eimeria* genus and species identity from faecal samples found to contain more than 500 oocysts per gram, ensuring recovery of DNA quantities appropriate for routine amplification (Kumar et al., 2014).

#### 2.2.3 Data collection

A standardised questionnaire was administered at each selected farm in parallel with faecal sample collection. The questionnaire comprised questions related to farm management, performance figures, bird characteristics, chicken health status and social factors associated with coccidiosis. A copy of the questionnaire is available from the corresponding author upon request. Most of the information in the questionnaire was completed based on the farm records maintained by each farmer. Questions related to unit characteristics (e.g. type of bedding, birds’ contact with faeces, water supply and fly infestation) were completed by the surveyor during farm observation.

### 2.3 Data analysis

All statistical analyses were performed in R 3.1.1 using packages epicalc and FactoMiner. Descriptive statistics, stratified by region, were obtained for management practices and farm characteristics captured in the questionnaires. The proportion of farms positive for any *Eimeria* species and each *Eimeria* species were calculated using Microsoft Excel within Windows version 7. P values <0.05 were considered to be significant.

#### 2.3.1 Identification of risk factors and univariate analysis

Putative risk factors assessed here included bird age and breed, flock size and purpose, management variables including the type of unit, feed and litter, biosecurity measures such as use of disinfection on entry, waste management system, bird access to faecal material, frequency of waste removal and fly infestation, distance to the closest poultry farm and access to the general public. The inclusion of dietary anticoccidials was considered a putative protective factor (where data were available). Data on age and flock size were collected as continuous data. Flock size was subsequently categorised based on quartiles (≤450, 451–1100, 1101–4000 and >4000 in the north and 1–300, 301–2000, 2001–3125 and >3125 in the south), and flock age was categorised as young (less than 8 weeks) and adult (greater than eight weeks). The age ranges were chosen since susceptibility to clinical coccidiosis is more common in birds less than eight weeks old, primarily as a consequence of limited prior parasite exposure and thus incomplete immune protection (Williams, 1998). Chicken breed was re-categorised as indigenous to India (Aseel and Kadaknath), non-indigenous commercial breeds or hybrids (those imported from other countries or including crossbreds developed in other countries: Ross 308, Babcock, Bovans, Rhode Island Red, Rhode Island White, White leghorn, Cobb and Cobb-100) and indigenous-commercial crossbreds (arising from a cross between indigenous and non-indigenous commercial breeds, developed in India: Wencobb, Gramalxmi, Grampriya, Karinga brown and BV-300). Bedding was categorised into natural (paddy husk, sawdust, wooden slatted or earth) and artificial (wired/caged), since birds maintained on natural bedding were more likely to be exposed to oocysts as a consequence of faecal exposure (Lunden et al., 2000).

Initially, the extent to which predictor variables associated with the presence of *Eimeria* or specific *Eimeria* species was assessed using univariate logistic regression to calculate odds ratios (OR) as an indication of risk of detection if a certain variable or category of variable was present. Subsequently, collinearity was explored using the Chi-square test for those variables identified as significant with a p-value ≤0.05 in the univariate analysis.

#### 2.3.2 Multivariate analysis

Multivariate analysis was performed to describe farm profiles based on management practices and farm characteristics. However, some variables (type of feed, access to the public, type of unit and waste management) were not considered either due to missing data or because a variable measuring similar factors was included. Multiple correspondence analysis (MCA) was performed in order to transform correlated variables into a smaller number of synthetic uncorrelated factors. Hierarchical cluster analysis (HCA) was then used to group farms into clusters according to their level of similarity with respect to those factors created by the MCA. The Euclidean distance was used to assess the level of dissimilarity between two farms. The algorithm was agglomerative and Ward’s criteria for linkage were used.

Odds ratios were obtained as a measure of strength of association between clusters and the presence of *Eimeria* species using Wald’s test to determine significance of association. *Eimeria* species were categorised into different pathogenic levels as described previously (Fornace et al., 2013). Specifically, *E. necatrix* was considered to be very highly pathogenic, *E. brunetti* and *E. tenella* were highly pathogenic, *E. acervulina* and *E. maxima* medium, with *E. mitis* and *E. praecox* primarily presenting a low pathogenicity risk.

## 3 Results

### 3.1 Parasite occurrence

**Fig. 2:**
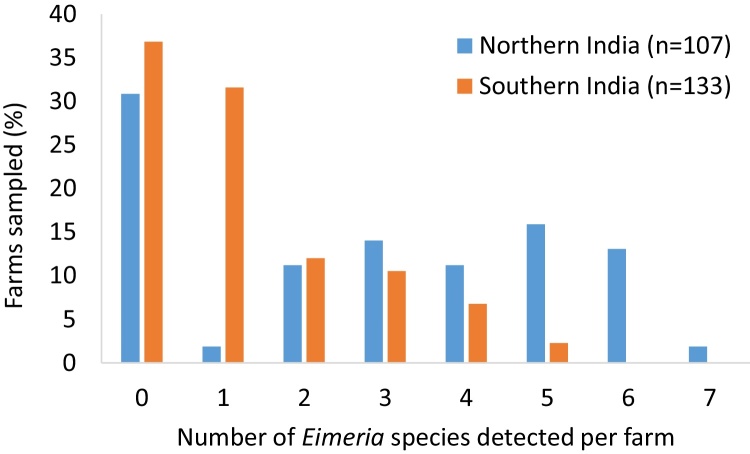
Number of *Eimeria* species identified per farm in northern and southern India.

In the present study 79.4% (85/107) and 76% (101/133) of farms from northern and southern India were found to be positive for any *Eimeria* by oocyst microscopy and genus-specific PCR. All *Eimeria* species were detected in north India, and only *E. praecox* was undetected in the south (Table 1). *Eimeria tenella*, followed by *E. mitis*, were the most common species detected in both sampled regions. *Eimeria acervulina* and *E. necatrix* were also common, with the former ranking higher in the north and the latter in the south (Table 1). The ranking of species occurrence was comparable between regions (rank correlation 0.86, p < 0.05). For all species, occurrence was lower in the south than the north, and co-infection with more than one species was more common in northern India (p < 0.05, Chi^2^ test; Fig. 2).

### 3.2 Univariate analysis of putative risk factors associated with *Eimeria* occurrence

In northern India bird age and breed type was associated with *Eimeria* occurrence, with younger birds and non-indigenous commercial breeds more likely to be positive (Table 2). In southern India such association was not detected, with similar occurrence for both age categories and indigenous as well as non-indigenous birds. Flock purpose was also found to influence the detection of *Eimeria*, with layer (north and south) and multi-purpose birds less likely to be positive than broilers. A small number of broiler breeder flocks were sampled in south India, although these were sampled within the same age range as the commercial broilers. Some management practices exerted a significant effect on *Eimeria* detection. Use of natural rather than artificial litter type and the removal of waste during the flock rearing period associated with a higher likelihood of *Eimeria* occurrence, but free-range production and the use of scavenging or feeding household waste was linked with reduced occurrence. Public access was related to reduced occurrence, while use of farm entrance disinfectant increased detection of *Eimeria*. In the south only access to faeces (absence of wire or gridded floors), flock purpose and distance to the closest farm were found to significantly associate with the presence of *Eimeria* species (Table 2).

### 3.3 Multivariate analysis of farm profiles associated with *Eimeria* occurrence

#### 3.3.1 Identification of farm profiles

Three clusters were identified following HCA in both northern and southern India that were loosely related to broiler, layer and indigenous birds kept for multi- or mixed broiler/layer purposes respectively (Table 3). Cluster 1 in northern India (broiler-N) comprised of farms with non-indigenous commercial breeds, primarily broilers (92%), and good cleaning practices (66% of the farms reported that they removed waste routinely and approximately 70% disinfected the farm and unit entrance). Crossbred commercial/indigenous broiler type birds were not encountered in northern India. In the south, cluster 1 (broiler-S) comprised largely of broiler and a small number of broiler breeder chickens of commercial and crossbred genotypes. All broiler-S farms reported that they disinfect farm and unit entrances routinely. Removal of the minority broiler breeder subset did not change the outcome of the analysis. Cluster 2 in the north (layer-N) included all farms which used artificial materials for bedding and mostly reared commercial or commercial-hybrid birds, the majority of which were layers (91%). Cluster 2 in the south (layer-S) comprised entirely of layers that were kept in cages and had no direct contact with their faeces. Cluster 3 in the north comprised entirely of small backyard farms, almost all of which stocked indigenous breeds which were used for multiple purposes (indigenous-N). Similarly, cluster 3 in the south included only small farms, most of which kept indigenous or crossbred commercial/indigenous birds for meat and/or egg production (indigenous-S; Table 3). About 97% of broiler-N farms were positive for *Eimeria* (Table 4). A higher proportion of farms in all clusters in northern India were positive to the most pathogenic species (*E. necatrix*) compared to clusters in south India. A similar pattern was observed for *E. tenella*, with 80% of broiler-N farms positive.

#### 3.3.2 Farms profiles associated with *Eimeria* occurrence

Odds ratios associated with the likelihood of occurrence of *Eimeria* of different pathogenicity groups were calculated for northern and southern India using the predominantly broiler cluster from each region as the arbitrarily chosen reference group (Table 5). Farms in the north Indian layer-N and indigenous-N clusters were less likely to be positive for *Eimeria* compared to broiler-N for any *Eimeria*, while indigenous-N farms were also less likely to be positive for each *Eimeria* pathogenicity group. In southern India the only significant association was detected for the indigenous-S cluster (small farms keeping indigenous birds), which was more likely to include farms positive for very high pathogenic *Eimeria* (odds ratio 2.91, P = 0.036; Table 5).

## 4 Discussion

The study reported here revealed considerable diversity in the distribution of *Eimeria* species according to poultry unit size, system and management practice within and between northern and southern India. Cluster analysis defined three distinct farm groups that were common to each region, permitting comparative analysis. These represent three major strands of poultry production, comprising broilers (including broiler breeders), layers, and indigenous or crossbred commercial/indigenous birds kept for multiple purposes (Table 3). A similar proportion of units were positive for *Eimeria* parasites in northern and southern India, but the occurrence of every individual *Eimeria* species was higher in northern India, commonly with a greater complexity of infection per unit. *Eimeria praecox* was not detected in southern India, although recent reports have previously found this species to be present in both northern and southern India (Aarthi et al., 2010, Kumar et al., 2015). The differences in the current study between northern and southern Indian poultry sectors could reflect greater organisation in the south, leading to higher awareness among farmers regarding biosecurity and disease control measures (Karthikeyan and Nedunchezhian, 2013), although this was not borne out by the univariate analysis. Video surveillance and modelling of chicken movements within poultry systems could be used to improve understanding of pathogen flow (Srinivasa Rao et al., 2015). The climate also varied between regions. *Eimeria* occurrence is known to vary as a consequence of season (Deichmann and Eklundh, 1991), with greater humidity associating with higher litter oocyst numbers, possibly a consequence of improved oocyst sporulation and survival. However, we did not find a higher number of farms to be positive for *Eimeria* in the more humid southern region, again suggesting better control measures.

*Eimeria tenella* was the most common species detected in both northern and southern India. Previous genetic analysis of this parasite, including the samples assessed here, revealed a striking dichotomy in genome-wide haplotype diversity and population structure. *Eimeria tenella* samples from northern India were defined by lower haplotype complexity and significant linkage disequilibrium compared to southern populations (Blake et al., 2015). Here, the association between type of system and presence of *Eimeria* was different in northern and southern India. In the north, commercial broiler farms were at higher risk. In southern India this was not observed and although associations between type of farm and parasite occurrence were not significant (probably related to the small number of flocks in the layer and indigenous flock clusters as reflected by the wide 95% CIs), the trend was in the opposite direction with broilers at lower risk. Combined, these points provide strong evidence of heterogeneity in the relationship between type of farm and presence of *Eimeria* in the two regions. Thus, type of farm is important, but has a different impact in the north than in the south. For *E. tenella* the broader spread of occurrence across south Indian poultry clusters, and the limited *E. tenella* occurrence in north Indian backyard poultry, may contribute to the restricted divergence and interbreeding of this species in northern India, compared to much greater interbreeding in the south.

In the univariate analysis the failure of farm entrance disinfection to limit *Eimeria* occurrence was unsurprising since the environmental oocyst stage of the eimerian life cycle is resistant to many commonly used disinfectants (Lunden et al., 2000). In the north we found that farms holding birds less than eight weeks old were six times more likely to be positive for any *Eimeria* than older ones (p-value = <0.0001). This is in agreement with previous reports that found young birds more susceptible to *Eimeria* species and/or less likely to have become immune by previous exposure (Williams, 1995). Excretion of oocysts has also been reported to be higher in birds between 4 and 8 weeks of age (Long et al., 1975, Williams, 1998).

The correlation between predictor variables hampered assessment of the potential role of specific risk factors, since it was not possible to adjust for the effect of other predictor variables given the high collinearity between them. For example, units found to use artificial litter such as wire flooring were also highly likely to host laying birds which were fed manufactured feed. Similarly, the finding that non-indigenous commercial birds were more likely to be positive for *Eimeria* compared to indigenous and indigenous crossbred birds could be attributed to multiple possible variables including management system and/or chicken genetic variation (Henken et al., 1992). Thus, linkage between some variables precluded adjusted assessment of their impact as individual measures, although they remained valuable descriptors of each system. However, HCA analysis of each cluster was more informative. Farms within the north Indian broiler cluster were most likely to be infected with detectable levels of *Eimeria*. These farms tended to have middle to upper quartile flock sizes and 66% of them reported that they removed waste routinely (often at less than 30 day intervals). Intensive production of broiler chickens, commonly including relatively young immunologically naïve birds kept at high stocking densities in contact with their faeces, is well known to associate with *Eimeria* occurrence since such conditions promote faecal-oral pathogen transmission (Williams, 1998, Lunden et al., 2000). Frequent removal of waste during flock cycles may exacerbate oocyst transmission as a consequence of litter disturbance.

North Indian layer and indigenous farm clusters were at significantly lower risk of *Eimeria* occurrence than northern broilers. The exception was *E. necatrix*, where risk was not significantly different. In northern India 43% of samples were positive for *E necatrix*, in contrast with just 15% in southern India. The high pathogenicity and economic cost associated with this parasite indicates greater risk of losses to north Indian poultry farmers (Fornace et al., 2013). More surprisingly, farms in the north Indian layer cluster were at a higher risk for many pathogenicity groups than those in the northern indigenous cluster. The northern layer cluster primarily included chickens that were kept in cages and did not have any contact with faeces. Nonetheless, they were still found to frequently excrete oocysts, possibly a consequence of time kept on litter while young and subsequent low level recycling, hindering the development of robust immune protection (Williams, 1998).

In southern India, the broiler flock cluster was at the lowest risk of being positive to any *Eimeria*, although this was not statistically significant. This cluster comprised of broilers and broiler breeders. Half of the breeder farms reported anticoccidial vaccination using the live vaccine Livacox Q™ (13/85 farms in CS-1). Vaccination would have been expected to increase circulating oocyst occurrence during the sampling period (Williams, 1998), but this was not reflected in the odds of infection in this cluster compared to other clusters. Livacox Q™ includes attenuated lines of *E. acervulina*, *E. maxima*, *E. necatrix* and *E. tenella*. Thus, while anticoccidial vaccine administration might have been expected to increase oocyst occurrence, the low reproductive capacity of the vaccinal oocysts may have resulted in limited representation within subsequent *Eimeria* field populations. The prophylactic use of anticoccidial drugs was only recorded in north India, where no significant impact on *Eimeria* occurrence was detected. This result may reflect the presence of anticoccidial resistant parasite strains, or the use of ionophorous anticoccidials which permit low level oocyst cycling. The only significant difference among clusters in south India was an increased odds of *E. necatrix* occurrence in indigenous cluster flocks, for which none of the representatives used anticoccidial vacination. Thus, southern backyard farms were at higher risk of harbouring *E. necatrix* than commercial broiler, broiler breeder and layer farms. It is possible that factors such as scavenging and free range management were responsible for this, although the longer pre-patent period and lower fecundity associated with this parasite species might also be important (Long et al., 1976).

The results of this study should be interpreted with caution as it had some limitations. Most of the commercial broiler farms sampled reared birds under contract with either Venketeshwara Hatcheries Private Ltd. or Suguna Poultry Farms Ltd. However, these two companies dominate broiler production in the regions sampled and do represent the majority chicken populations. Nonetheless, the data collected contained sufficient information to support the analysis presented here. The strong correlation between multiple individual management factors requires either a very large cross-sectional study or a cohort study with purposive selection of farms exposed or not to the risk factor of interest to be able to assess the relationship between these practices and infection. Further, the presence of eimerian oocysts in faeces is not always associated with economic losses (Fornace et al., 2013). Variation in pathogenicity between parasite species, and strains can lead to varied flock-level disease (Long et al., 1976, Williams et al., 2009). Similarly, the use of ionophorous anticoccidials reduces, but does not eradicate oocyst production, providing an opportunity for development of protective immunity whilst limiting *Eimeria* replication (Chapman et al., 2010). Thus, the impact of *Eimeria* is attributable largely to the presence of highly pathogenic species combined with the magnitude of challenge, particularly to young birds that have not developed immunity.

In this study we have classified poultry production into three systems and found that different *Eimeria* occurrence was associated with different types of farms. Notably, we have detected an uneven occurrence of the highly pathogenic species *E. necatrix*, featuring in many north Indian flocks. Considerable variation was also determined for pathogenic species such as *E. tenella*, providing a possible driver for the polymorphism in genetic variation and population structure described previously. As demand for poultry products increases in India it is likely to be mirrored by a surge in poultry production and intensification. As the chicken population increases so too does the risk of infectious disease among chickens, which may in turn be reflected by changes in population structure and genetic diversity defining the pathogens. Understanding and responding to the challenges in pathogen control which may arise as a result of such changes will require ongoing surveillance and assessment. Knowledge defining the differences between poultry system clusters can improve understanding of *Eimeria* evolution, and may prove relevant to the maintenance of future drug or vaccine based control strategies.

## Figures and Tables

**Table 1:** The relative pathogenicity and occurrence (number and ranking) of *Eimeria* species detected on poultry farms sampled in northern and southern India. na = not applicable. Rankings 1–7 indicates most to least common.

*Eimeria* species	Pathogenicity group	North (n = 107)		South (n = 133)	
		No. positive (%)	Rank	No. positive (%)	Rank
Any *Eimeria* species	–	85(79.4)	na	101(76.0)	na
*Eimeria necatrix*	Very high	46 (43.0)	4	20 (14.9)	3
*Eimeria brunetti*	High	4 (3.7)	7	1 (0.7)	6
*Eimeria tenella*	High	72 (67.3)	1	77 (57.5)	1
*Eimeria acervulina*	Medium	49 (45.8)	3	17 (12.7)	4
*Eimeria maxima*	Medium	30 (28.0)	6	12 (9.0)	5
*Eimeria mitis*	Low	63 (58.9)	2	40 (29.9)	2
*Eimeria praecox*	Low	35 (32.7)	5	0 (0.0)	7

**Table 2:** Explanatory variables for the presence of any *Eimeria* with count, number and percentage of positives (Positive), Odds ratio (OR), 95% Confidence interval (95% CI) and P value of Wald’s test for the univariate association with positive status. CB = indigenous/non-indigenous crossbred. *not used in MCA. NA refers to variables for which there was insufficient data to conduct the analysis. OR of 1 indicates the category used as the base line for comparison within this variable.

Variables	Northern India					Southern India				
	Category (n)	Positive (%)	OR	95% CI	P	Category (n)	Positive (%)	OR	95% CI	P
Age group of birds	Young (n = 74)	66 (89.2)	6.08	(2.22−16.65)	<0.001	Young (n = 64)	47 (73.4)	0.84	(0.39,1.82)	0.658
	Adult (n = 33)	19 (57.6)	1	–	–	Adult (n = 69)	55 (76.4)	1	–	–
Breed category of birds	Indigenous (n = 23)	13 (56.5)	1	–	–	Indigenous (n = 35)	29 (83)	1.4	(0.5,3.96)	0.522
	Indigenous CBs (n = 8)	4 (50)	5	(0.15,3.86)	0.75	Indigenous CBs (n = 27)	16 (59.3)	0.41	(0.16,1.05)	0.064
	Non-indigenous (n = 60)	52 (86.7)	10.77	(1.65,15.18)	0.005	Non-indigenous (n = 71)	57 (78)	1	–	–
Size of the flock	≤450 (n = 28)	16 (57.1)	0.39	(0.11,1.36)	0.141	≤300 (n = 35)	29 (82.9)	1	–	–
	451–1100 (n = 25)	24 (96)	7.06	(0.76,65.95)	0.087	301–2000 (n = 40)	31 (77.5)	0.71	(0.23,2.25)	0.564
	1101–4000 (n = 31)	27 (87.1)	1.99	(0.47,8.45)	0.353	2001–3125 (n = 26)	19 (70.4)	0.49	(0.15,1.64)	0.248
	>4000 (n = 22)	17 (77.3)	1	–	–	>3125 (n = 33)	24 (68.6)	0.45	(0.15,1.4)	0.169
Type of litter material	Natural (n = 92)	77 (83.7)	3.84	(1.31,11.26)	0.014	Natural (n = 124)	98 (78.4)	2.9	(0.73,11.57)	0.131
	Artificial (n = 15)	8 (53.3)	1	–	–	Artificial (n = 9)	5 (55.6)	1	–	–
Type of feed given*	Scavenging/Household waste (n = 24)	12 (50)	0.14	(0.05,0.39)	<0.001	Scavenging (n = 18)	15 (83.3)	1.96	(0.53,7.28)	0.315
	Manufactured feed (n = 83)	73 (87.9)	1	–	–	Manufactured feed (n = 109)	78 (71.5)	1	–	–
Fly infestation	Yes (n = 21)	18 (85.7)	1.7	(0.45,6.4)	0.431	NA				
	No (n = 86)	67 (78)	1	–	–					
Access to faeces (no wire floor)	Yes (n = 80)	66 (82.5)	1.98	(0.72,5.44)	0.182	Yes (n = −122)	97 (78.9)	4.97	(1.59,15.61)	0.006
	No (n = 27)	19 (70.4)	1	–	–	No (n = 11)	6 (42.9)	1	–	–
Access to public*	Yes (n = 27)	15 (55.6)	0.18	(0.07,0.49)	<0.001	Yes (n = 74)	57 (24)	1.33	(0.58,3.07)	0.506
	No (n = 80)	70 (87.5)	1	–	–	No (n = 41)	31 (70.5)	1	–	–
Distance to closest farm	100-2000metres (n = 68)	55 (81)	1	–	–	No other local unit (n = 35)	32 (74.4)	1	–	–
	Up to 100 m (n = 28)	21 (75)	0.71	(0.25,2.02)	0.52	Up to 100 m (n = 42)	38 (91.4)	0.27	(0.07,1.07)	0.063
	No other local unit (n = 11)	9 (82)	1.06	(0.2,5.52)	0.941	100-2000metres (n = 56)	32 (65.4)	0.18	(0.05,0.65)	0.009
Flock purpose	Broiler (n = 61)	59 (95.2)	1	–	–	Breeder (n = 30)	25 (83.3)	1	–	–
	Layer (n = 22)	14 (63.6)	0.09	(0.02,0.38)	0.001	Broiler (n = 75)	61 (71.3)	0.89	(0.29,2.72)	0.832
	Multi-purpose (n = 21)	11 (47.6)	0.05	(0.01,0.2)	<0.001	Layer (n = 28)	16 (75)	0.21	(0.06,0.7)	0.011
Type of unit*	Completely enclosed (n = 59)	49 (83.5)	1	–	–	Completely enclosed (n = 115)	88 (74)	0.61	(0.16,2.26)	0.458
	Free range (n = 28)	17 (60.7)	0.32	(0.11,0.87)	0.026	Free range (n = 17)	14 (82.4)	1	–	–
	Open sided, roofed (n = 19)	19 (100)	1	–	–					
Waste management*	Removed (n = (91)	78 (85.7)	7.71	(2.44,24.34)	<0.001	Removed (n = 51)	39 (72.2)	0.85	(0.39,1.88)	0.69
	Never removed (n = 16)	7 (43.8)	1	–	–	Never removed (n = 76)	57 (75)	1	–	–
Frequency of waste removal	≤30 days (n = 55)	49 (89)	1	–	–	NA				
	30–60 days (n = 14)	12(85.7)	0.75	(0.13,4.19)	0.596					
	≥60 days (n = 37)	23(62.1)	0.67	(0.15,2.98)	0.743					
Farm entrance disinfected	Yes (n = 67)	58 (67.5)	3.1	(1.18,8.14)	0.021	Yes (n = 55)	42 (75)	0.98	(0.45,2.16)	0.967
	No (n = 40)	27 (86.5)	1	–	–	No (n = 78)	61 (75.4)	1	–	–
Coccidiostat given	Yes (n = 57)	49 (86)	2.38	–	–	NA				
	No (n = 50)	36 (72)	1	(0.9,6.28)	0.079					
Vaccination against coccidiosis	NA					Yes (n = 16)	15 (93.8)	5.62	(0.71,44.28)	0.101
						No (n = 117)	88 (72.7)	1	–	–

**Table 3:** Characteristics of poultry farms belonging to clusters identified by hierarchical cluster analysis of 107 farms in northern India (N) and 133 farms in southern India (S). CB = indigenous/non-indigenous crossbred. Clusters, primarily representing large scale broiler farms (= broiler), large scale layer farms (layer) and small scale & backyard farms (indigenous).

Variables	Northern India				Southern India			
	Category (n)	Broiler-N (n = 60)	Layer-N (n = 22)	Indigenous-N (n = 25)	Category (n)	Broiler-S (n = 85)	Layer-S (n = 9)	Indigenous-S (n = 39)
		Number (%)	Number (%)	Number (%)		Number (%)	Number (%)	Number (%)
Age group of birds	Young (n = 74)	57 (77)	6 (8.1)	11 (14.9)	Young (n = 64)	60 (94)	0 (0)	4 (6)
	Adult (n = 33)	3 (9.1)	16 (48.5)	14 (42.4)	Adult (n = 69)	25 (36.2)	9 (13)	35 (51)
Breed category of birds	Indigenous (n = 23)	0 (0)	1 (4.4)	22 (95.6)	Indigenous (n = 35)	4 (11.4)	1 (2.8)	30 (86)
	Indigenous CBs (n = 8)	0 (0)	8 (100)	0 (0)	Indigenous CBs (n = 27)	16 (59.3)	4 (14.8)	7 (25.9)
	Non-indigenous (n = 60)	51 (85)	7 (11.7)	2 (3.3)	Non-indigenous (n = 71)	65 (83.5)	4 (9.5)	2 (6.8)
Size of the flock	≤450 (n = 28)	4 (14.3)	0 (0)	24 (85.7)	≤300 (n = 34)	1 (3)	0(0)	33 (97)
	451–1100 (n = 25)	24 (96)	1 (4)	0 (0)	301–2000 (n = 40)	29 (72.5)	5 (12.5)	6 (15)
	1101–4000 (n = 31)	27 (87.1)	4 (13)	0 (0)	2001–3125 (n = 26)	25 (96)	1 (4)	0 (0)
	>4000 (n = 22)	5 (22.7)	17 (77.3)	0 (0)	>3125 (n = 33)	30 (91)	3 (9)	0 (0)
Type of litter material	Natural (n = 98)	60 (68.2)	3 (3.4)	25 (28.4)	Natural sources (n = 124)	85 (68.5)	0 (0)	39 (31.5)
	Artificial (n = 19)	0 (0)	19 (100)	0 (0)	Artificial materials (n = 9)	0 (0)	9 (100)	0 (0)
Type of feed given	Scavenging/Household waste (n = 24)	0 (0)	0 (0)	24 (100)	Scavenging (n = 18)	0 (0)	0 (0)	18 (100)
	Manufactured feed (n = 83)	60 (72.3)	22 (26.5)	1 (1.2)	Manufactured feed (n = 99)	69 (69.7)	9 (9)	21 (21.3)
Fly infestation	Yes (n = 21)	5 (23.8)	9 (42.9)	7 (33.3)	Yes (n = 1)	0 (0)	0 (0)	1 (100)
	No (n = 86)	55 (64)	13 (15)	18 (21)	No (n = 132)	85 (64.4)	9 (6.8)	38 (28.8)
Access to faeces (no wire floor)	Yes (n = 80)	56 (70)	0 (0)	24 (30)	Yes (n = −122)	85 (69.7)	0 (0)	37 (30.3)
	No (n = 27)	4 (14.8)	22 (81.5)	1 (3.7)	No (n = 11)	0 (0)	9 (82)	2 (18)
Access to public	Yes (n = 27)	8 (29.6)	0 (0)	19 (70.4)	Yes (n = 74)	38 (51.3)	5 (6.7)	31 (42)
	No (n = 80)	52 (65)	22 (27.5)	6 (7.5)	No (n = 41)	29 (70.3)	4 (9.7)	8 (19.5)
Distance to closest farm	100-2000metres (n = 68)	43 (63.2	18 (26.5)	7 (10.3)	100-2000metres (n = 56)	37 (66)	8 (14.4)	11 (19.6)
	Up to 100 m (n = 28)	12 (42.9)	0 (0)	16 (57.1)	Up to 100 m (n = 42)	17 (40.5)	0 (0)	25 (59.5)
	No other local unit (n = 11)	5 (45.5)	4 (36.4)	2 (18.2)	No other local unit (n = 35)	31 (88.6)	1 (2.8)	3 (8.6)
Flock purpose	Broiler (n = 61)	57 (92)	0 (0)	5 (8)	Breeder (n = 30)	29 (96.6)	0 (0)	1 (3.3)
	Layer (n = 22)	2 (9.1)	20 (91)	0 (0)	Broiler (n = 75)	49 (65.3)	0 (0)	26 (34.7)
	Multi-purpose (n = 21)	1 (4.8)	1 (4.8)	19 (90.5)	Layer (n = 28)	7 (25)	9 (32)	12 (43)
Type of unit	Completely enclosed (n = 59)	42 (71.2)	16 (27)	1 (1.7)	Completely enclosed (n = 115)	84 (73)	9 (7.8)	22 (19.2)
	Free range (n = 28)	3 (10.7)	2 (7.1)	23 (82)	Free range (n = 17)	0 (0)	0 (0)	17 (100)
	Open sided, roofed (n = 19)	15 (79)	3 (15.8)	1(5.2)				
Removal of waste	Removed (n = 91)	60 (66)	22 (24.2)	9 (9.8)	Removed (n = 51)	42 (82.3)	0 (0)	9 (17.6)
	Never removed (n = 16)	0 (0)	0 (0)	16 (100)	Never removed (n = 76)	41 (54)	9 (12)	26 (34)
Frequency of waste removal	≤30 days (n = 55)	41 (74.5)	8 (14.5)	6 (10.9)	NA			
	30–60 days (n = 14)	10 (71.4)	4 (28.6)	0 (0)				
	≥60 days (n = 38)	9 (23.7)	10 (26.3)	19 (50)				
Farm entrance disinfected	Yes (n = 67)	48 (71.6)	19 (28.4)	0 (0)	Yes (n = 55)	50 (91)	4 (7.2)	1 (1.8)
	No (n = 40)	12 (30)	3 (7.5)	25 (62.5)	No (n = 78)	35 (44.8)	5 (6.5)	38 (48.7)
Coccidiostat given	Yes (n = 57)	41 (72)	15 (26.3)	1 (1.7)	NA			
	No (n = 50)	19 (38)	7 (14)	24 (48)				
Vaccination against coccidiosis	NA				Yes (n = 16)	13 (81.3)	0(0)	3 (18.7)
					No (n = 117)	72 (61.5)	9 (7.7)	36 (30.8)

**Table 4:** The relative pathogenicity and occurrence (number and proportions shown for each) of *Eimeria* species detected in different clusters from northern (N) and southern (S) India. Clusters, primarily representing large scale broiler farms (= broiler), large scale layer farms (layer) and small scale & backyard farms (indigenous).

*Eimeria* species	Pathogenicity group	North (n = 107)			South (n = 133)		
		Broiler-N (n = 60)	Layer-N (n = 22)	Indigenous-N (n = 25)	Broiler-S (n = 85)	Layer-S (n = 9)	Indigenous-S (n = 39)
Any *Eimeria* species	–	58(96.7)	14(63.6)	13(52.0)	65(76.5)	5(55.6)	31(79.5)
*Eimeria necatrix*	Very high	27(45.0)	8(36.4)	9(36.0)	8(9.4)	3(33.3)	8(20.5)
*Eimeria brunetti*	High	4(6.7)	0(0.0)	1(4.0)	1(1.2)	0(0.0)	0(0.0)
*Eimeria tenella*	High	48(80.0)	13(59.0)	9(36.0)	49(57.6)	5(55.6)	23(59.0)
*Eimeria acervulina*	Medium	34(56.7)	8(36.4)	6(24.0)	16(18.8)	0(0.0)	1(2.5)
*Eimeria maxima*	Medium	23(38.3)	1(4.5)	4(16.0)	9(10.6)	1(11.1)	2(5.1)
*Eimeria mitis*	Low	41(68.3)	11(50.0)	10(40.0)	24(28.2)	3(33.3)	13(33.3)
*Eimeria praecox*	Low	23(38.3)	7(31.8)	4(16.0)	0(0.0)	0(0.0)	0(0.0)

**Table 5:** Odds ratios (OR), 95% confidence interval (95% CI) and P value (statistical significance, Wald’s test) for association with the presence of any, or specific pathogenic categories of *Eimeria* species and poultry production HCA clusters relative to large scale broiler farms (selected arbitrarily as the baseline to permit comparison; cluster names representative of the majority system-type within each cluster as shown in Table 3). Odds more than double or less than half that of the broiler cluster are highlighted in red or green respectively, with their associated P values highlighted and underlined in the same colour where significant. (For interpretation of the references to color in this table legend, the reader is referred to the web version of this article.)
